# Safety of chronic hypertonic bicarbonate inhalation in a cigarette smoke-induced airway irritation guinea pig model

**DOI:** 10.1186/s12890-022-01919-x

**Published:** 2022-04-07

**Authors:** Kata Csekő, Dóra Hargitai, Lilla Draskóczi, Adrienn Kéri, Pongsiri Jaikumpun, Beáta Kerémi, Zsuzsanna Helyes, Ákos Zsembery

**Affiliations:** 1 Department of Pharmacology and Pharmacotherapy, Medical School, University of Pécs, Pécs, 7624 Hungary; 2 Molecular Pharmacology Research Group, Szentágothai Research Centre, Pécs, 7624 Hungary; 3 2nd Department of Pathology, Semmelweis University, Budapest, 1091 Hungary; 4 Department of Oral Biology, Faculty of Dentistry, Semmelweis University, Nagyvárad tér 4, Budapest, 1089 Hungary; 5 Heim Pál Children Hospital, Budapest, 1089 Hungary; 6 Department of Conservative Dentistry, Faculty of Dentistry, Semmelweis University, Budapest, Hungary; 7 PharmInVivo Ltd, Pécs, 7629 Hungary

**Keywords:** Airways, Cystic fibrosis, COPD, Bicarbonate, pH, Inhalation

## Abstract

**Background:**

Cystic fibrosis (CF) and chronic obstructive pulmonary disease (COPD) are often associated with airway fluid acidification. Mutations in the cystic fibrosis transmembrane conductance regulator (*CFTR*) gene leads to impaired bicarbonate secretion contributing to CF airway pathology. Chronic cigarette smoke (CS) -the major cause of COPD- is reported to induce acquired CFTR dysfunction underlying airway acidification and inflammation. We hypothesize that bicarbonate-containing aerosols could be beneficial for patients with CFTR dysfunctions. Thus, we investigated the safety of hypertonic sodium bicarbonate (NaHCO_3_) inhalation in CS-exposed guinea pigs.

**Methods:**

Animals were divided into groups inhaling hypertonic NaCl (8.4%) or hypertonic NaHCO_3_ (8.4%) aerosol for 8 weeks. Subgroups from each treatment groups were further exposed to CS. Respiratory functions were measured at 0 and after 2, 4, 6 and 8 weeks. After 8 weeks blood tests and pulmonary histopathological assessment were performed.

**Results:**

Neither smoking nor NaHCO_3_-inhalation affected body weight, arterial and urine pH, or histopathology significantly. NaHCO_3_-inhalation did not worsen respiratory parameters. Moreover, it normalized the CS-induced transient alterations in frequency, peak inspiratory flow, inspiratory and expiratory times.

**Conclusion:**

Long-term NaHCO_3_-inhalation is safe in chronic CS-exposed guinea pigs. Our data suggest that bicarbonate-containing aerosols might be carefully applied to CF patients.

**Supplementary Information:**

The online version contains supplementary material available at 10.1186/s12890-022-01919-x.

## Background

Cystic fibrosis (CF) is a fatal hereditary condition caused by mutations in the *cystic fibrosis conductance regulator* (CFTR) gene. Although it is a multiorgan disorder, morbidity and mortality are attributed to progressive airway complications, exhibited as chronic obstructive lung disease. Over the last decades, chronic respiratory diseases (CRDs) such as chronic obstructive pulmonary disease (COPD), asthma and bronchiectasis have become leading causes of morbidity and mortality worldwide. Despite the different pathogeneses, COPD and CF share common phenotypic features, such as airflow limitation, mucus obstructions and progressive deterioration of pulmonary function [1]. Impaired mucus clearance leads to repeated lung infections and may contribute to the chronicity of COPD, thus physiological airway functions are closely correlated to epithelial ion and water transport [2]. Airway acidification has been shown in both CF and COPD [3, 4], which could be due to defective bicarbonate (HCO_3_^−^) transport through the CFTR anion channel [5]. In fact, CFTR^−/−^ pigs exhibit reduced airway pH and impaired bacterial killing, which are increased after aerosolizing NaHCO_3_ into the trachea [6]. While CF is relatively rare, other CRDs affect hundreds of millions of people worldwide. Importantly, growing body of evidence suggests that acquired CFTR dysfunction underlies chronic rhinosinusitis, COPD, non-atopic asthma, non-CF bronchiectasis and tobacco smoke-induced pulmonary diseases [7, 8]. It has been reported that cigarette smoke exposure, the major cause of COPD, leads to downregulation of CFTR mRNA, protein and function [9–11] and CFTR activity is reduced in smokers both with and without COPD associated with chronic bronchitis and the severity of dyspnea [12]. Such an acquired CFTR-dysfunction can also reduce the mucociliary clearance and may contribute to COPD pathogenesis [3, 13]. Furthermore, uncompensated proton secretion tends to further acidify the airways which could be an important pathogenic factor in CRDs [14]. Ivacaftor, and GLPG2196 CFTR potentiators have already been shown to reverse cigarette smoke extract-induced CFTR-dysfunctionin vitro and COPD ferrets as well, respectively [15, 16]. Therefore, drugs developed to enhance CFTR activity might also be beneficial in COPD patients [17].

Bicarbonate acts not only as a buffer, but it has many other important roles in the airways. Impaired bicarbonate secretion is likely to be responsible for aggregated mucus in CF mice [18] and pigs [19]. Re-administration of bicarbonate reduces mucus viscosity and corrects mucociliary transport [18]. These effects are especially important because viscous mucus and impaired mucociliary transport provide an appropriate environment for pathogen growth, evoking immune response and inflammation. We have recently found that NaHCO_3_ inhibits both the growth and biofilm formation of bacteria relevant in CF [20, 21]. Moreover, in order to mimic sodium bicarbonate inhalation treatment, apical administration of 75 mM HCO_3_^−^-containing media to CF bronchial epithelial cells was also well-tolerated, suggesting that these cells can endure changes in tonicity, pH and HCO_3_^−^ [22]. Furthermore, HCO_3_^−^ alters bacterial susceptibility to antibiotics [23] and oral NaHCO_3_ activates splenic anti-inflammatory pathways [24].

Hypertonic saline nebulization has long been used as a mucolytic treatment for CF [25]. Animal studies also demonstrated the potential of hypertonic saline in alleviating mucus obstruction in a spontaneous lung disease model using βENaC transgenic mice exhibiting airway surface dehydration characteristic to CF and COPD [13]. However, the above-mentioned data suggest that inhalation of HCO_3_^−^-containing aerosols might be an even more effective therapeutic approach in CF and/or CRDs. It is no accident that usage of inhalation solution of mineral salt containing high amount of HCO_3_^−^ (5.6 g/l) is recommended for patients with rhinosinusitis, acute or chronic bronchitis, COPD, bronchial asthma and CF [26]. However, to our best knowledge, no in vivo animal data are available on the effects of long-term hypertonic NaHCO_3_ inhalation.

In light of the listed beneficial effects of HCO_3_^−^ as well as the cigarette smoke-induced acquired CFTR-deficiency we intended to investigate the impact of hypertonic NaHCO_3_ inhalation on general physiologic and respiratory parameters in a mild cigarette smoke-exposure model to mimic the molecular alterations characteristic to both CF and COPD. We have chosen this animal model because guinea pig lungs translate more to human airway pathophysiology [27]. On the other hand, CF mice do not exhibit lung disease presumably because of lacking ATP12A protein in the apical membrane of airway epithelial cells [3].

## Materials and methods

### Animals

Experiments were performed on 8-week-old male guinea pigs weighing 600 ± 150 g at the beginning of the study. Animals were bred and kept in the Laboratory Animal House of the Department of Pharmacology and Pharmacotherapy, University of Pécs, Hungary at 24–25 °C, provided with standard chow, vegetables and fruits and water ad libitum, maintained under 12 h light–dark cycle. All procedures were performed in accordance with the 40/2013 (II.14.) Government Regulation on Animal Protection and Consideration Decree of Scientific Procedures of Animal Experiments and Directive 2010/63/EU of the European Parliament. They were approved by the Animal Welfare Committee of the University of Pécs and the National Scientific Ethics Committee on Animal Research of Hungary (licence No.: BA02/2000–4/2019 issued on 29 Jan 2019 by the Government Office of Baranya County).

### Experimental design

Guinea pigs were divided into 4 groups (4 animals/group); 2 groups treated with hypertonic NaCl (8.4% corresponding to 1.44 M) and the other 2 groups with hypertonic NaHCO_3_ (8.4% corresponding to 1 M) aerosol for 30 min, twice daily, 5 days/week, for 8 weeks. Hypertonic NaCl and NaHCO_3_ solutions were prepared freshly each week and were aerosolized by a nebulizer (1–5 μM particle size**;** Boneco 7145 W ultrasonic nebulizer, BonAir BG Ltd., Budapest, Hungary) into 55 × 35 × 40 cm boxes where guinea pigs were placed during inhalational treatment. The treatment groups were subdivided into groups inhaling only NaCl or NaHCO_3_, and groups exposed to cigarette smoke besides the respective aerosol treatments. Cigarette smoke exposure (CSE) was performed after aerosol treatment in a whole-body smoke exposure chamber (Teague Enterprise, USA) for 30 min followed by a ventilation period of 30 min twice daily, 10 times/week for 8 weeks with the use of 2 research cigarettes at a time (3R4F Kentucky Research Cigarette; University of Kentucky, USA) [28]. Body weight was measured daily, respiratory functions were assessed at the beginning and at the end of week 2, 4, 6 and 8. At the end of the experimental protocol animals were anaesthetized by pentobarbital sodium (1% Euthanimal 400 mg/ml, Alfasan, the Netherlands; 0.5 ml/100 g) and arterial as well as venous blood was collected for laboratory tests. Lungs were excised and fixed in 6% formaldehyde solution for histopathological assessment.

### Investigation of respiratory functions

Airway function was measured by unrestrained whole-body plethysmography (WBP) (PLY3213 Buxco Europe Ltd., Winchester, UK) at the beginning and at the end of week 2, 4, 6 and 8 in conscious, spontaneously breathing guinea pigs. Breathing frequency, tidal volume, minute ventilation, inspiratory and expiratory times, peak inspiratory and expiratory flows, as well as baseline enhanced pause (Penh) correlating with airway resistance were measured for 15 min following a 15-min-long acclimation period.

### Histopathological evaluation

Lung samples were fixed in 6% paraformaldehyde solution and embedded in paraffin. Hematoxylin–eosin staining was performed on 5 μm sections for the assessment of lung pathophysiology. Three different localizations (apex, hilus, base) were excised from the lungs of each animal. Slides were examined using a bright field microscope (Olympus CH30). Ten non-cartilaginous airways, ten vessels and ten septa (examined by high power field) were selected from each lung site of every group (equally chosen from each animal). Acute inflammatory cell infiltration (eosinophil and neutrophil granulocytes) was counted in the airways, vessels and septa to evaluate the extent of airway inflammation [29]. Airway intraluminal perimeter was measured with Case Viewer software (3DHISTECH Ltd, Hungary) after scanning each slide with 20 × objective (Pannoramic 250 FLASH III scanner, 3DHistech Ltd., Hungary). Airway intraluminal perimeter was used to normalize airway dimensions.

### Laboratory parameters

Urine pH was assessed every week of the experimental protocol. Animals were placed in a metabolic cage for the period of urine collection. pH was assessed from freshly collected urine by a FiveEasyPlus™ pH meter (Mettler Toledo, Hungary). At the end of the experimental protocol arterial blood was collected for blood gas and acid base analysis by Astrup's equilibration technique in the Department of Laboratory Medicine, University of Pécs, Hungary. Other laboratory parameters were measured from heparinized venous blood by an AU5800 clinical chemistry analyzer (Beckman Coulter Hungary, Budapest, Hungary) in the Deparment of Laboratory Medicine, Semmelweis University, Budapest, Hungary.

### Statistical analysis

Statistical analysis was performed by GraphPad Prism v6 software (GraphPad, San Diego, CA, USA). Respiratory parameters and body weight were analyzed by repeated measures two-way ANOVA followed by Tukey's multiple comparisons test. Histopathological and laboratory parameters were assessed by Kruskal–Wallis followed by Dunn's multiple comparisons test.

## Results

### Long-term NaHCO_3_ inhalation improves some CSE-induced transient respiratory alterations

**Fig. 1:**
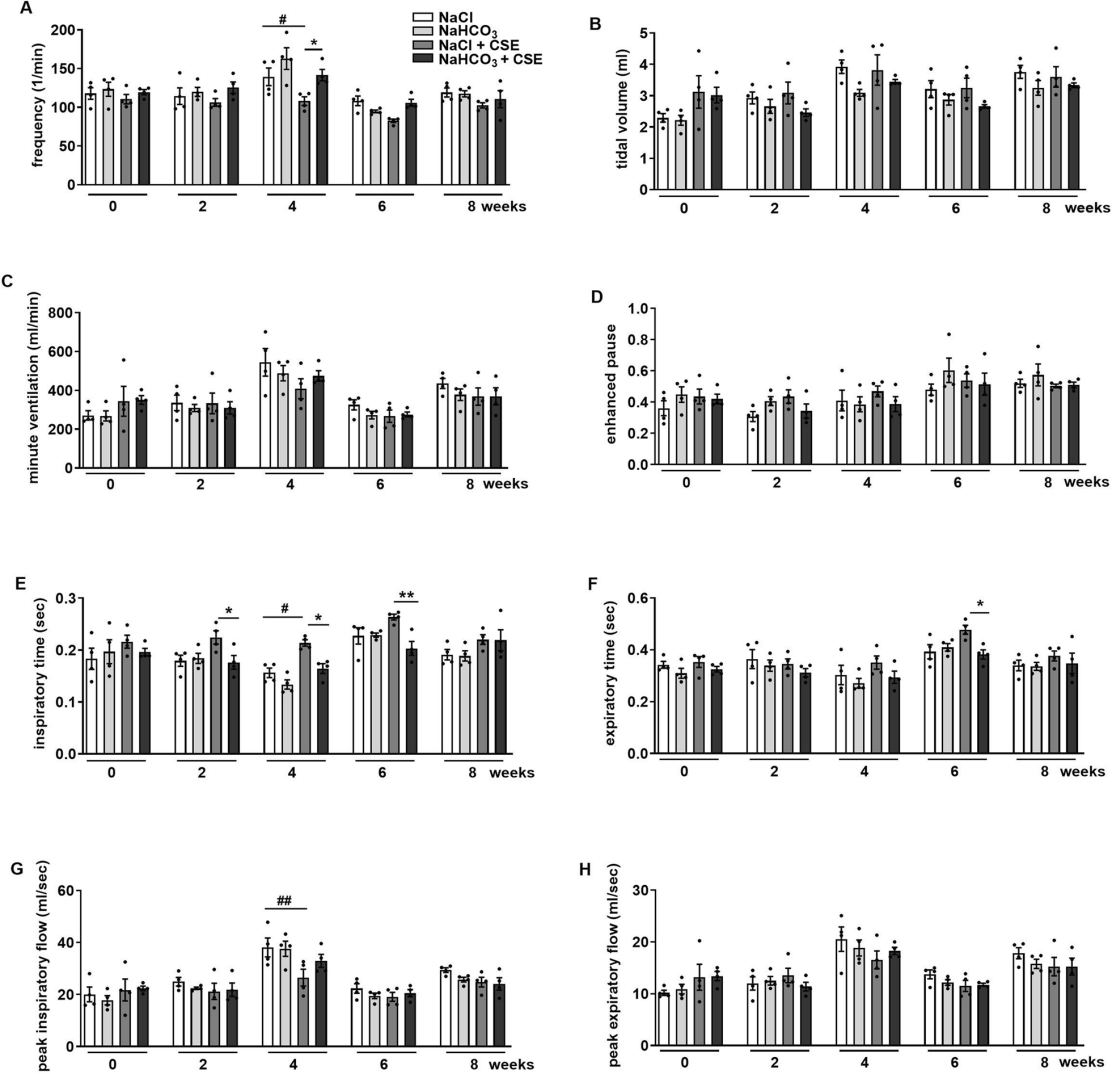
Respiratory function parameters, such as (**a**) frequency, (**b**) tidal volume, (**c**) minute ventilation, (**d**) enhanced pause, (**e**) inspiratory time, (**f**) expiratory time, (**g**) peak inspiratory flow, (**h**) peak expiratory flow measured by unrestrained whole body plethysmography. n = 4/group, data represent means ± SEM, repeated measures two-way ANOVA followed by Tukey's multiple comparisons test *p < 0.05, **p < 0.005 vs. NaCl + CSE-treated group; #p < 0.05 ##p < 0.05 vs. NaCl-treatment

Frequency, inspiratory and expiratory times, as well as peak inspiratory flow showed mild and transient significant alterations in response to CSE throughout the 8-week-long experimental protocol. In the 8.4% NaCl + CSE-treated group frequency and peak inspiratory flow significantly decreased, while inspiratory time increased compared to the non-smoking respective controls at the end of week 4 (Fig. 1a,c,e,g). These alterations were counteracted by 8.4% NaHCO_3_ treatment in CS-exposed animals. The protective effect of NaHCO_3_ is also supported by the facts that i) in contrast to the NaCl-treated animals, no significant differences developed in any parameters of the NaHCO_3_ + CSE-treated guinea pigs in comparison with the respective controls, and ii) the inspiratory and expiratory times were significantly shorter in the NaHCO_3_ + CSE group compared to the NaCl + CSE animals at weeks 2 and 6 (Fig. 1e, f). There were no changes in tidal volume, Penh and peak expiratory flow in any groups (Fig. 1b,d,h). At the end of the treatments no differences were revealed in the parameters of different experimental groups. The parameters were similar to intact conditions (Additional file 1: Fig. 1), thus long-term hypertonic NaHCO_3_ aerosol inhalation did not induce any respiratory functional deteriorations in either group.

### Histopathological changes

**Fig. 2:**
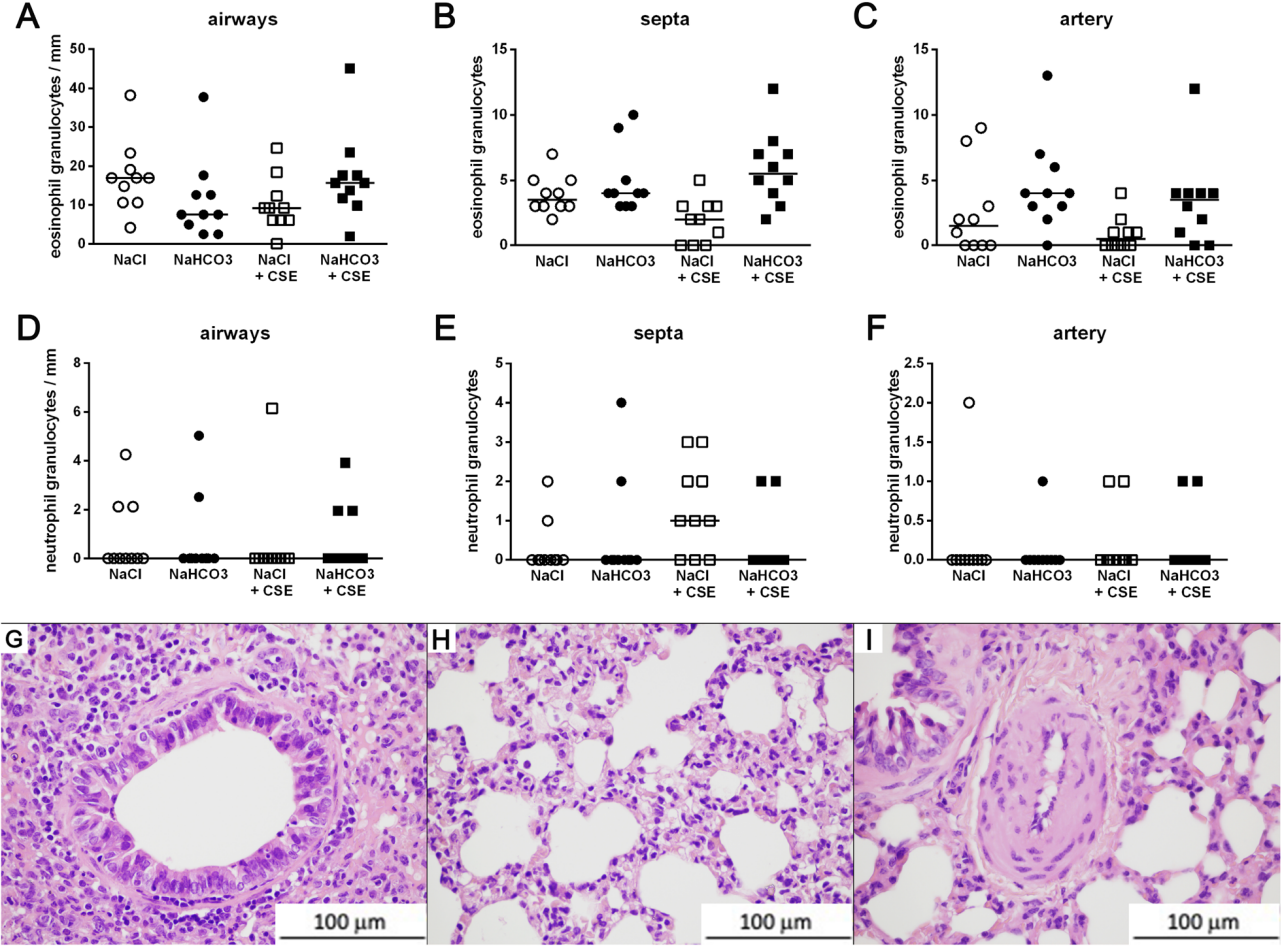
Histopathological assessment of inflammatory cells in the lung. Eosinophil (**a-c**) and neutrophil (**d-f**) granulocyte numbers measured in the airways (**a,d,g**), septa (**b,e,h**) and vessels (**c,f,i**). n = 10 measurements group, graph represents individual data and median; Kruskal–Wallis, followed by Dunn's multiple comparisons test

**Fig. 3:**
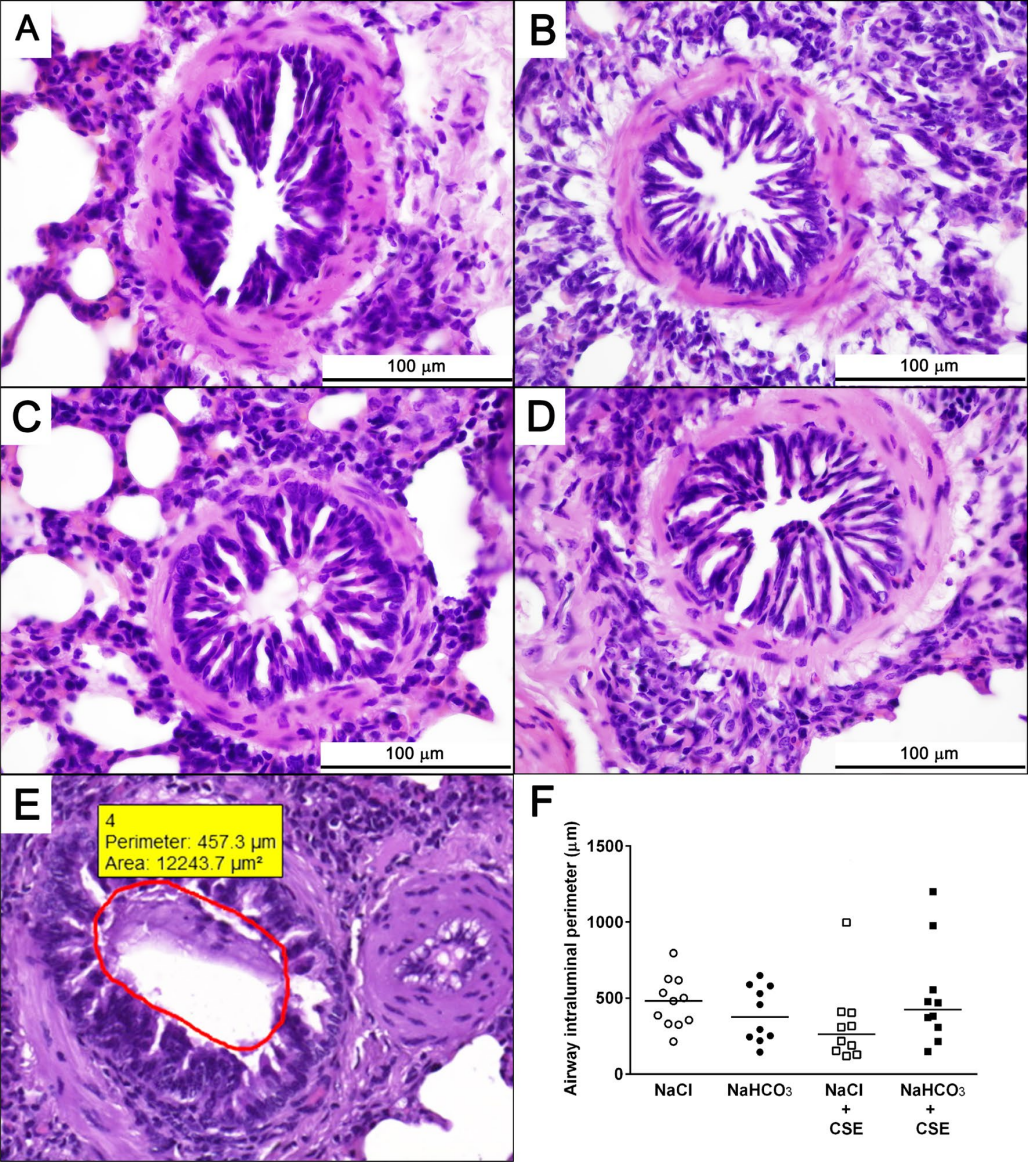
Assessment of airway intraluminal perimeter. Representative histopathological pictures of bronchioles of (**a**) NaCl, (**b**) NaHCO_3_, (**c**) NaCl + CSE, and (**d**) NaHCO_3_ + CSE-treated guinea pigs. Neither treatment induced significant changes in airway intraluminal perimeter (**f**). n = 10 measurements/group, graph represents individual data and median; Kruskal–Wallis followed by Dunn's multiple comparisons test. Panel (**e**) represents the method for measurement

Chronic hypertonic NaHCO_3_ inhalation did not induce significant eosinophil (Fig. 2a–c) or neutrophil granulocyte infiltration (Fig. 2d–f) measured in the non-cartilaginous airways, vessels and septa of lung (Fig. 2g–i). There was no decreased airway intraluminal perimeter (Fig. 3f) either in the non-smoking (Fig. 3a, b) or CS-exposed (Fig. 3c,d) groups quantified by the Case Viewer software (3DHISTECH Ltd, Hungary) (Fig. 3e). There were some lymphoid follicles sporadically observed in the lung sections of NaCl-, NaCl + CSE-, as well as NaHCO_3_-treated guinea pigs. For representative histopathological pictures see Additional file 1: Fig. 2.

### Laboratory parameters

**Fig. 4:**
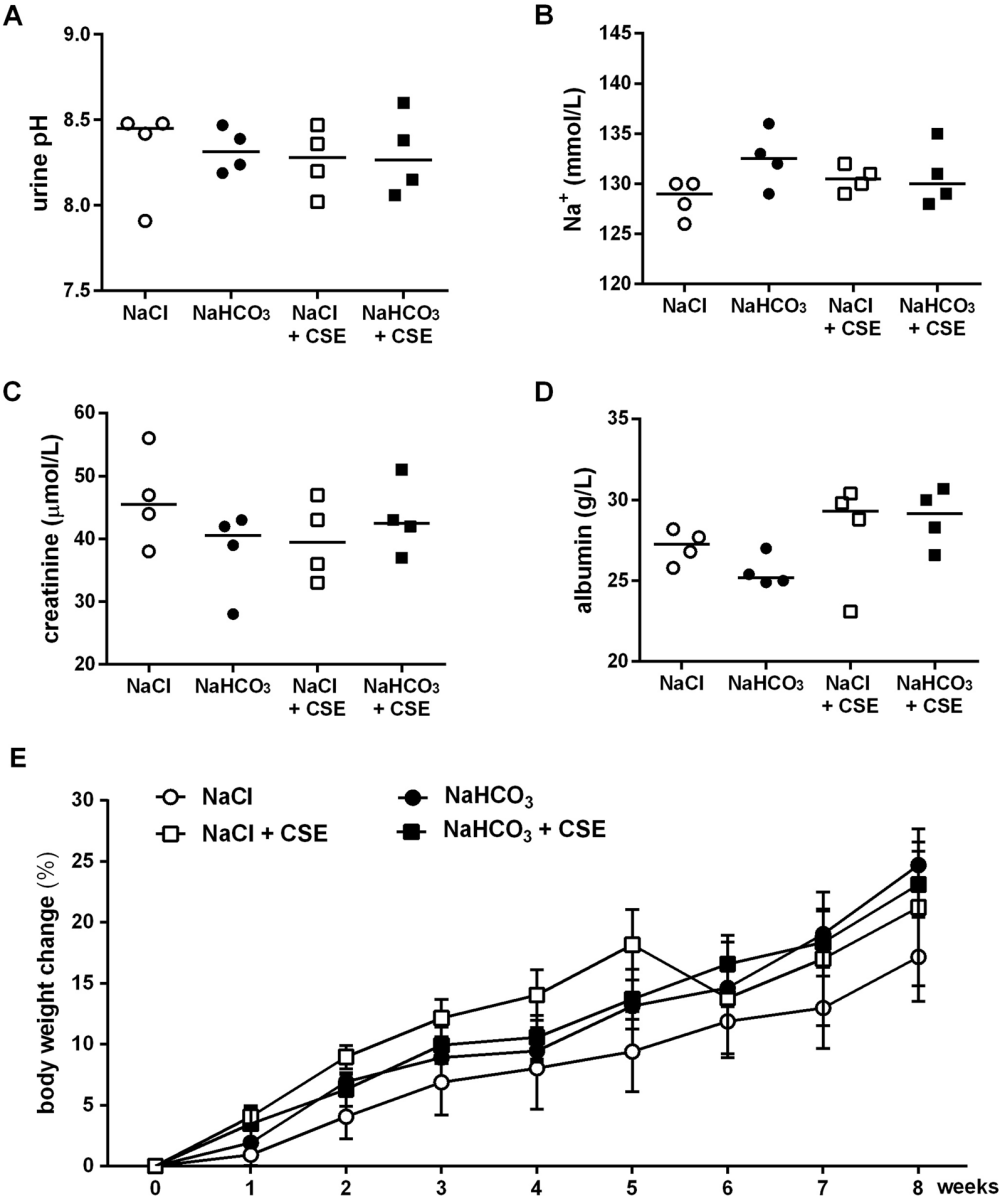
Laboratory parameters and body weight change at the end of the treatment protocol. Urine pH (**a**), sodium (Na +) (**b**), creatinine (**c**) and albumin levels (**d**) of guinea pigs 8 weeks after hypertonic bicarbonate aerosol and chronic cigarette smoke exposure compared to sodium chloride treatment. Graph represents individual data and median; Kruskal–Wallis followed by Dunn's multiple comparisons test. Panel (**e**) demonstrates weight change throughout the 8-week-long protocol. Data represent means ± SEM, two-way ANOVA followed by Tukey's multiple comparisons test

Parameters of electrolyte balance, such as sodium and chloride; creatinine referring to kidney and albumin, total protein, bilirubin, alkaline phosphatase (ALP) as well as alanine transaminase (ALT) levels indicating liver functions were within the normal range (Fig. 4 and Table 1). Hyperkalemia observed in all groups might be due to hemolysis upon blood collection. Chronic inhalation of hypertonic NaHCO_3_ did not induce metabolic alkalosis, the alkaline urine pH characteristic of herbivores was within the physiologic range. The arterial blood gas analysis performed before tissue harvesting revealed acute respiratory acidosis with elevated P_a_CO_2_ and acidotic arterial pH that could be most likely due to pentobarbital anaesthesia-induced respiratory depression.

**Table 1:** Laboratory parameters after hypertonic bicarbonate aerosol and cigarette smoke exposure compared to sodium chloride treatment

Parameter	NaCl	NaHCO_3_	NaCl + CSE	NaHCO_3_ + CSE
Arterial pH	7.17	7.35 ± 0.22	7.23	7.16 ± 0.07
standard HCO_3_ (mmol/l)	17.1	26.9 ± 5.85	19.1	18.1 ± 1.52
PaCO_2_ (mmHg)	64.3	59.5 ± 10.45	59	72.83 ± 4.61
Glucose (mmol/l)	6.08 ± 0.31	6.87 ± 0.26	7.21 ± 0.54	7.35 ± 0.29
Triglyceride (mmol/l)	1.32 ± 0.21	1.27 ± 0.29	1.38 ± 0.08	1.40 ± 0.44
Total bilirubin (mmol/l)	1.10 ± 0.11	1.00 ± 0.32	0.78 ± 0.05	0.83 ± 0.19
Direct bilirubin (mmol/l)	0.33 ± 0.05	0.40 ± 0.17	0.23 ± 0.05	0.25 ± 0.06
ALT (IU/l)	71.68 ± 5.89	77.35 ± 4.63	66.60 ± 7.58	65.23 ± 6.71
AST (IU/l)	103.33 ± 21.08	85.25 ± 6.52	127.55 ± 16.81	118.55 ± 22.00
GGT (IU/I)	8.85 ± 2.28	6.48 ± 0.59	7.55 ± 0.61	7.70 ± 0.33
ALP (IU/l)	73.25 ± 4.11	71.75 ± 7.44	68.50 ± 7.40	68.50 ± 7.40
LDH (IU/l)	140.75 ± 21.55	128.25 ± 38.94	169.25 ± 21.78	142.50 ± 13.58
Cholesterol (mmol/l)	0.78 ± 0.17	0.82 ± 0.09	0.80 ± 0.05	0.93 ± 0.13
Amylase (IU/l)	2304.5 ± 126.70	2114.50 ± 132.35	2270.25 ± 128.72	2497.75 ± 136.27
Lipase (IU/I)	31.50 ± 14.65	18.50 ± 5.20	15.50 ± 0.65	25.25 ± 8.50
Total Protein (g/l)	55.23 ± 0.54	51.90 ± 0.99	52.55 ± 2.41	56.78 ± 1.98
Urea (mmol/l)	10.73 ± 0.46	10.18 ± 0.89	10.00 ± 1.25	11.28 ± 0.99
K^+^ (mmol/l)	9.19 ± 1.15	8.38 ± 1.37	8.47 ± 0.37	7.48 ± 0.77
Cl^−^ (mmol/l)	94.00 ± 0.41	92.25 ± 1.93	96.00 ± 0.58	94.25 ± 0.85
Ca^2+^ (mmol/l)	2.89 ± 0.05	2.66 ± 0.07	2.77 ± 0.06	3.00 ± 0.04
Mg^2+^ (mmol/l)	1.26 ± 0.07	0.98 ± 0.06	1.09 ± 0.04	1.14 ± 0.06
P (mmol/l)	2.39 ± 0.43	1.97 ± 0.28	2.12 ± 0.08	2.19 ± 0.18
Fe (mmol/l)	36.40 ± 2.91	38.10 ± 2.78	41.10 ± 3.82	46.28 ± 5.95

Data represent mean ± SEM of 4 animals/group. PaCO_2_: ALT: alanine transaminase, AST: aspartate transaminase, GGT: gamma-glutamyl-transpeptidase, ALP: alkaline phosphatase, LDH: lactate dehydrogenase, K + : potassium, Cl-: chloride, Ca2 + : calcium, Mg2 + : magnesium, P: phosphorus, Fe: iron

The body weight gain of the animals was affected neither by hypertonic NaHCO_3_ aerosol treatment nor by CSE (Fig. 4e).

## Discussion

Although sodium bicarbonate has been considered as a mucolytic agent for decades, there is no consensus about its usefulness in mucus clearance disorders [26]. Patients with CF and other CRDs instill natural inhalation solutions (i.e. spring waters of mineral salts), often without doctor’s recommendation. Although the composition of these solutions is different, high HCO_3_^−^ concentrations (up to approx. 178 mM corresponding to 1.5%) is their common feature. Furthermore, a number of recent evidence suggests that HCO_3_^−^ has not only mucolytic activity. It reduces inflammatory responses [24], inhibits bacterial growth and biofilm formation [20], enhances bacterial killing capacity of the innate immune system [6] and strengthens the efficacy of aminoglycosides [23] as well. Since all these effects would be desirable in CRDs, instillation of HCO_3_^−^ on the airways could be of versatile remedy. Thus, there is an urgent need to define both beneficial and possible harmful effects of chronic administration of bicarbonate-containing aerosols in vivo. Data presented here provide evidence that 8-week-long inhalation of either hypertonic sodium bicarbonate (8.4%) or sodium chloride (8.4%) does not elicit harmful effects even in cigarette smoke-exposed guinea pig airways, and therefore both may be considered to be equally safe. Furthermore, some cigarette smoke-induced mild respiratory functional changes are improved compared to hypertonic sodium chloride (8.4%) treatment.

There are different animal models of CRDs such as CF mice, ferrets and pigs [30], as well as cigarette smoke-exposed mice, rats and guinea pigs of COPD [28, 29, 31]. However, CF ferret and pig models are not only expensive but also difficult to breed, whereas CF mice do not develop CF lung disease [32]. Therefore, we chose the cigarette smoke-exposed guinea pig model considering that these animals are easy to breed and their lung anatomy and physiology share common features with humans [31]. Our data could have important implications in CF as well, since recent studies suggest substantial overlap between COPD and CF due to CFTR dysfunction [1, 2, 11, 12, 17, 33]. Hypertonic NaCl has long been used in the treatment of CF and is known to improve lung function and to have marked benefits regarding exacerbations [25, 34–36]. The primary goal of this study was to investigate the safety of hypertonic sodium bicarbonate inhalation in vivo*.* Cigarette smoke-exposure COPD models suffer a disadvantage because only mild airway disease develops in the first few months [29]. Indeed, our study has limitations, since 8 week-long protocol has not promoted significant airway inflammation. In fact, we cannot exclude the possibility that the lack of any signs of airway inflammation might have been masked by the beneficial effects of the hypertonic NaCl or NaHCO_3_, which prevented CS-induced alterations. However, extended and more intense exposure is required to induce substantial inflammation and consequent airway remodelling in this experimental paradigm [29]. Another caveat regarding inhalation therapy is that bicarbonate may deposit in upper airways potentially reducing either its efficacy or possible side effects. Nonetheless, mathematical models would be needed to describe the deposition of inhaled particles based on their size and the anatomical distribution of the bronchial tree [37].

Since hypertonic sodium chloride (3–7%) instillation is a well-established medication for CF patients [25], we have tested the most concentrated sodium bicarbonate solution (8.4%) available for injection [38]. Nebulized sodium bicarbonate solution (4.2%) has been shown to have beneficial short-term effects in patients with reactive airway dysfunction syndrome [39]. Bronchoalveolar lavage with NaHCO_3_ is safe and inhibits bacterial and fungal growth in patient with lower respiratory tract infection[40]. Recently, we have demonstrated that hypertonic (300 mmol/L) NaHCO_3_ reduces the gel strength of CF sputum samples [41]. Furthermore, nebulized NaHCO_3_ (both 4.2% and 8.4%) was found safe and well tolerated in the management of CF patients [42].

Our data show that neither blood nor urine pH increased in HCO_3_^−^-treated animals indicating that long-term inhalation of 8.4% NaHCO_3 _has not induced systemic alkalosis. Furthermore, weight-gain was not different in any groups suggesting that both hypertonic NaCl and NaHCO_3_ have been well-tolerated. However, it should be emphasized that administration of aerosols containing high concentrations of NaHCO_3_ might induce unpredictable changes in tonicity, pH and volume of ASL in vivo.

Mucus hypersecretion has been implicated in both CF and COPD pathology [43]. Furthermore, it has been demonstrated that cigarette smoke induced MUC5AC mucus overproduction which further highlights its potential involvement in airway pathogenesis [44, 45]. Interestingly, as opposed to COPD, studies assessing CF airway secretions reported rather controversial results of MUC5AC and MUC5B mucins [46, 47]. However, it is beyond doubt that mucus hypersecretion is a key feature in the airway pathology. Guaifenesin has been shown to inhibit MUC5AC significantly, as opposed to other mucolytics as N-acetylcysteine and ambroxol (48). Therefore, a plausible additional beneficial effect of hypertonic NaCl and NaHCO_3_ inhalation might be the potential inhibition of mucus production, which should also be addressed in further studies.

## Conclusion

We have demonstrated that 8-week-long inhalation of hypertonic NaHCO_3_ is as safe as NaCl in cigarette smoke-induced airway irritation guinea pig model. HCO_3_^−^ should therefore be considered a potentially valuable therapeutic agent in chronic inflammatory airway diseases.

## Supplementary Information

**Additional file 1.** **Figure 1.** Respiratory function parameters after 8-week-long treatment with hypertonic NaCl or hypertonic NaHCO_3_ aerosol compared to intact guinea pigs. Long-term NaCl and NaHCO_3_ inhalational treatment does not alter respiratory parameters. **Figure 2.** Representative histopathological pictures of HE-stained lung sections of guinea pigs after 8-week long treatment with hypertonic NaCl, NaHCO_3_, NaCl+CSE and NaHCO_3_+CSE.

## Data Availability

The datasets used and/or analysed during the current study are available from the corresponding author on reasonable request.

## Abbreviations

CF: Cystic fibrosis
CFTR: Cystic fibrosis transmembrane conductance regulator
COPD: Chronic obsructive pulmonary disease
CRD: Chronic respiratory disease
CS: Cigarette smoke
CSE: Cigarette smoke exposure
NaCl: Sodium chloride
NaHCO_3_: Sodium bicarbonate
