# Supplementary material for: Safety of chronic hypertonic bicarbonate inhalation in a cigarette smoke-induced airway irritation guinea pig model
Source: BMC Pulm Med. 2022 Apr 7;22:131. doi: 10.1186/s12890-022-01919-x (PMC8991956; doi:10.1186/s12890-022-01919-x)
Supplement: Supplementary file 1 — Additional file 1. Figure 1. Respiratory function parameters after 8-week-long treatment with hypertonic NaCl or hypertonic NaHCO3 aerosol compared to intact guinea pigs. Long-term NaCl and NaHCO3 inhalational treatment does not alter respiratory parameters. Figure 2. Representative histopathological pictures of HE-stained lung sections of guinea pigs after 8-week long treatment with hypertonic NaCl, NaHCO3, NaCl+CSE and NaHCO3+CSE. [file 12890_2022_1919_MOESM1_ESM.docx]

Supplementary Material

BMC Pulmonary Medicine

**Safety of chronic hypertonic bicarbonate inhalation in a cigarette smoke-induced airway irritation guinea pig model**

**Kata Csekő, Dóra Hargitai, Lilla Draskóczi, Adrienn Kéri, Pongsiri Jaikumpun, Beáta Kerémi, Zsuzsanna Helyes, Ákos Zsembery**

Additional results

**Long-term NaCl and NaHCO_3_ inhalational treatment does not alter respiratory parameters**

Neither hypertonic NaCl nor hypertonic NaHCO_3_ inhalation for 8 weeks altered respiratory functions; breathing frequency, tidal volume, minute ventilation, inspiratory and expiratory times, peak inspiratory and expiratory flows were not significantly different from age-matched (4-month-old) intact guinea pigs (Additional Figure 1).



**Additional Figure 1.** **Respiratory function parameters after 8-week-long treatment with hypertonic NaCl or hypertonic NaHCO_3_ aerosol compared to intact guinea pigs.** n = 4-16/ group, data represent means ± SEM, one-way ANOVA followed by Dunnett's multiple comparisons test.


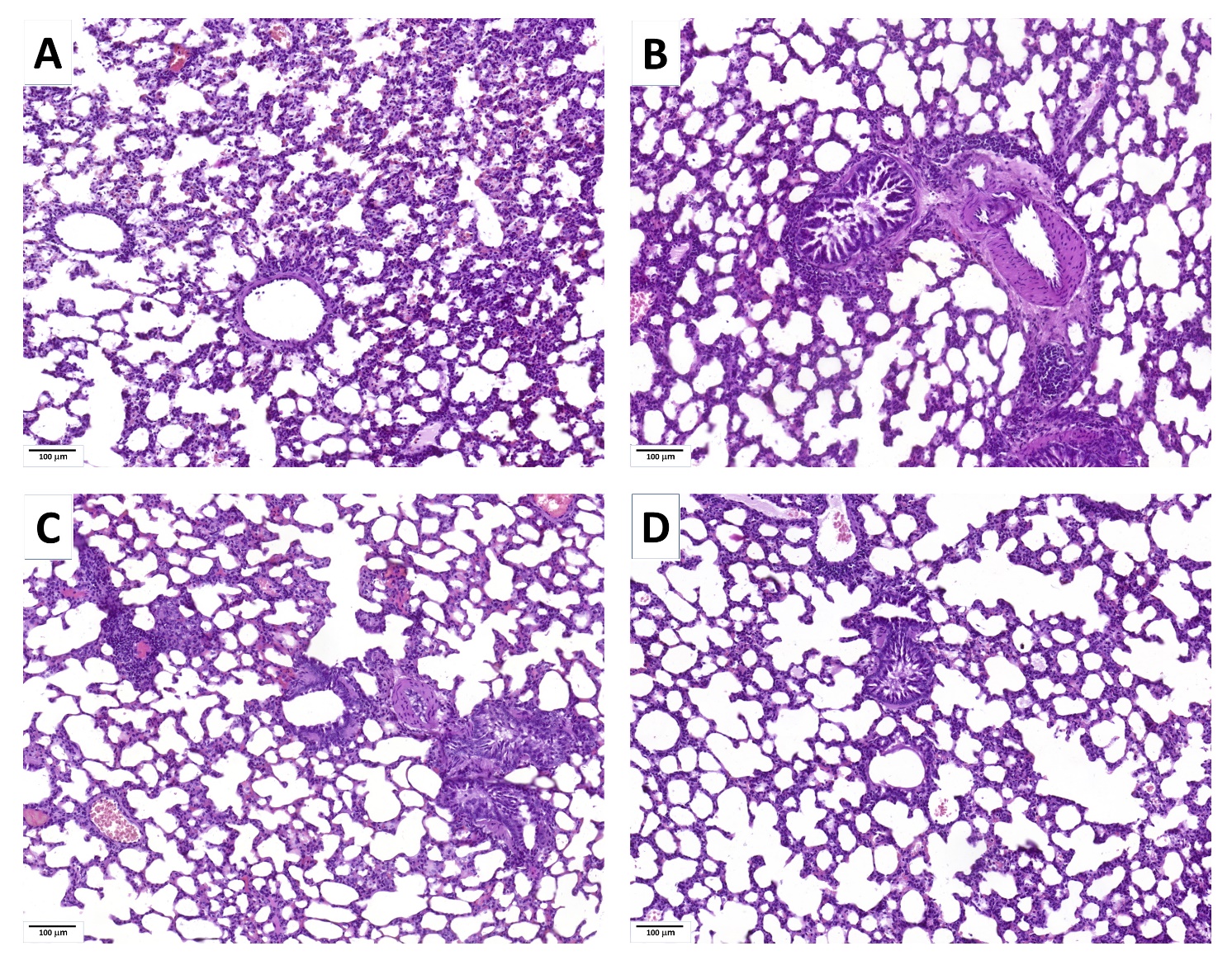


**Additional Figure 2. Representative histopathological pictures** of HE-stained lung sections of guinea pigs after 8-week long treatment with **(A)** NaCl, **(B)** NaHCO_3_, **(C)** NaCl+CSE, and **(D)** NaHCO_3_+CSE. (10x magnification)
